# BAF-Net: Bidirectional attention fusion network *via* CNN and transformers for the pepper leaf segmentation

**DOI:** 10.3389/fpls.2023.1123410

**Published:** 2023-03-27

**Authors:** Jiangxiong Fang, Houtao Jiang, Shiqing Zhang, Lin Sun, Xudong Hu, Jun Liu, Meng Gong, Huaxiang Liu, Youyao Fu

**Affiliations:** ^1^ Institute of Intelligent Information Processing, Taizhou University, Taizhou, Zhejiang, China; ^2^ Jiangxi Engineering Laboratory on Radioactive Geoscience and Big Data Technology, East China University of Technology, Nanchang, China; ^3^ Engineering Research Center of Development and Management for Low to Ultra-Low Permeability Oil & Gas Reservoirs in West China, Xi’an Shiyou University, Xi’an, China; ^4^ College of Mechanical Engineering, Quzhou University, Quzhou, Zhejiang, China

**Keywords:** convolution neural network, leaf segmentation, attention mechanism, multi-scale network, Swin Transformer

## Abstract

The segmentation of pepper leaves from pepper images is of great significance for the accurate control of pepper leaf diseases. To address the issue, we propose a bidirectional attention fusion network combing the convolution neural network (CNN) and Swin Transformer, called BAF-Net, to segment the pepper leaf image. Specially, BAF-Net first uses a multi-scale fusion feature (MSFF) branch to extract the long-range dependencies by constructing the cascaded Swin Transformer-based and CNN-based block, which is based on the U-shape architecture. Then, it uses a full-scale feature fusion (FSFF) branch to enhance the boundary information and attain the detailed information. Finally, an adaptive bidirectional attention module is designed to bridge the relation of the MSFF and FSFF features. The results on four pepper leaf datasets demonstrated that our model obtains F1 scores of 96.75%, 91.10%, 97.34% and 94.42%, and IoU of 95.68%, 86.76%, 96.12% and 91.44%, respectively. Compared to the state-of-the-art models, the proposed model achieves better segmentation performance. The code will be available at the website: https://github.com/fangchj2002/BAF-Net.

## 1 Introduction

Pepper is a common crop in China and has become an important vegetable and condiment in our daily life. However, pepper is a sensitive plant and pepper crops are highly exposed to diseases, which easily cause the frontal disease of the pepper leaves. The plant leaves can reflect plant growth, and pepper leaf diseases directly leads to the decline of pepper yield and quality. The visual characteristics of pepper leaf diseases is very similar, so it is not easy to distinguish them. With the advance of imaging technology, computer vision technologies have been widely used in plant leaf extraction to guide the agricultural expert to analyze the crop growth. By using image processing technology to analyze two-dimensional leaf image features, the plant growth stages could be dissected (Slaughter et al., 2008; Koirala et al., 2019), and monitor the plant diseases (Singh, 2019; Tian et al., 2019) by the analysis of the image various plant organs. Therefore, the accurate segmentation of pepper leaves from pepper images is of great significance for controlling pepper leaf diseases. However, it is challenging to design a general model for automatic segmentation of pepper leaves since the pepper leaves and some crops have similar phenotypic features (Hasan et al., 2021).

**Figure 1:**
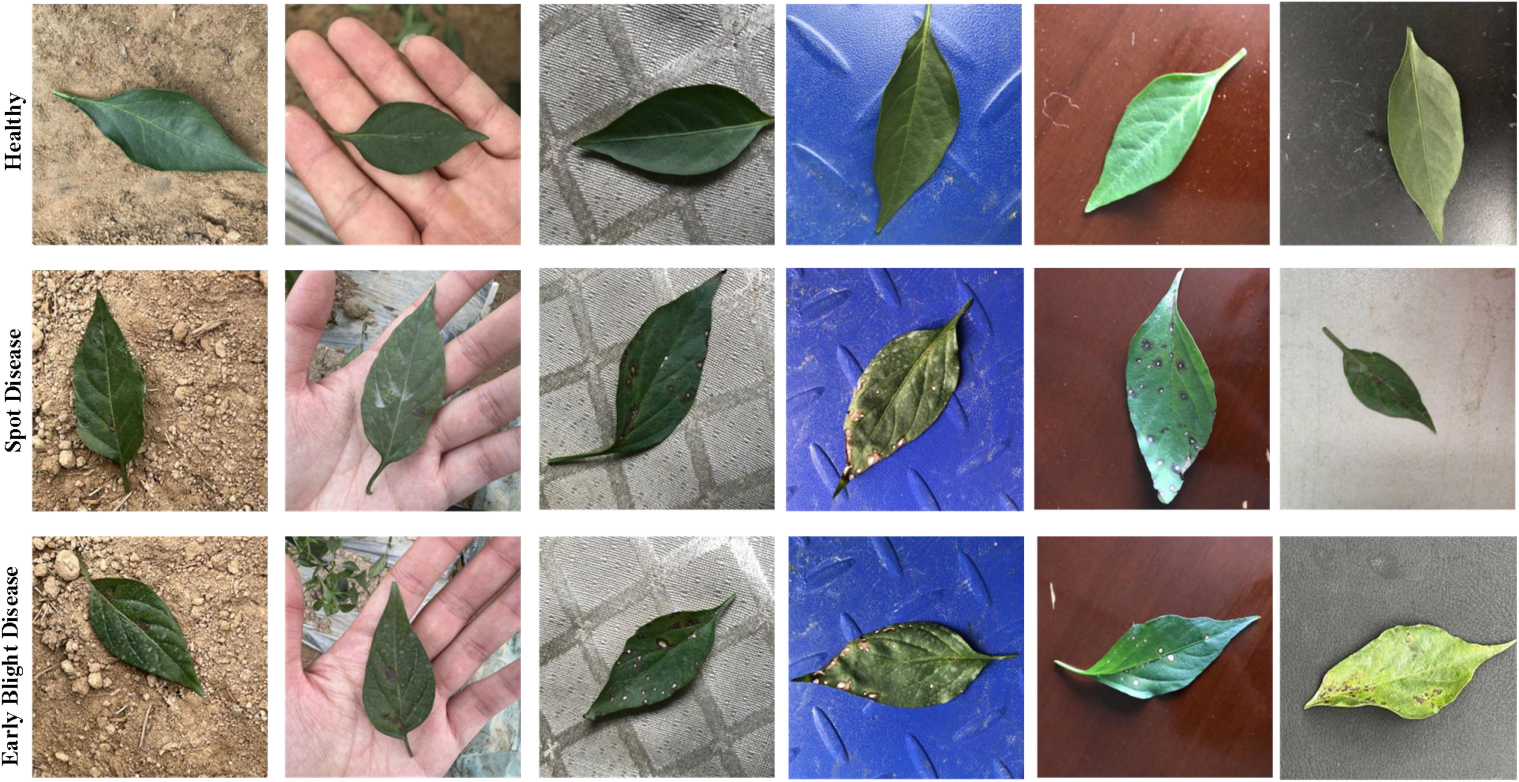
The sample dataset with different background.

Broadly speaking, the existing literature for the plant leaf segmentation can be classified into two categories as shown in **Figure 1**: conventional and deep learning-based methods. For the conventional methods, a statistical method with graph-based models (Kumar and Domnic, 2019) was proposed to segment the plant image and leaf counting, where the image enhancement techniques and the transformation from RGB to HSV were used to improve the quality of the input image. To avoid the problem of leaf over-segmentation, green channel information (Wang et al., 2018) was used to remove the background information, and the Sobel operator was improved to segment cucumber leaves. To detect the occluded plant leaves, leaf shape (Xia et al., 2013) was fused into the energy function to segment the leaf images. To deal with the complex background and the strong illumination, Larese et al. (2014) proposed a leaf vein analysis method for leguminous leaf segmentation and classification. The automatic segmentation method for plant leaf images under complex background was proposed to obtain the segmentation results. Scharr et al. (2016) uses the supervised classification with a neural network along with color and watershed transform for plant leaf segmentation and counting. Kuznichov et al. (2019) proposed a schema to augment the training dataset and remain the geometrical structure of the plant leaf by constructing a generation synthetic data. To segment multiple leaves at the same time and deal with the leaf over-segmentation, a deep extraction method for plant leaf (Amean et al., 2021) was proposed by incorporating multiple features, such as color, shape, and depth information. Lin et al. (Lin et al., 2023) proposed a self-supervised blade segmentation framework consisting of a self-supervised semantic segmentation model, a color-based blade segmentation algorithm, and a self-supervised color correction model. A self-supervised semantic segmentation model (Lin et al., 2023) was proposed to deal with the complex lighting conditions. The model was comprised of the features extracted from the CNN-based network and the fully connected Conditional Random Fields (CRFs), thus significantly reducing the impact of complex backgrounds and variations within the leaf and non-leaf regions.

In recent years, the deep learning-based method has outperformed the conventional segmentation methods and shows great potential in processing plant phenotypic tasks (Bhagat et al., 2021; Chandra et al., 2020). The SegNet-based model (Aich and Stavness, 2017) with the encoder-decoder architecture was used to segment plant leaves and leaf counting. Three RGB images and the segmentation mask of leaf counting were used as four input channels to build a regression model. Thus, the SegNet-based model can solve the problem of leaf counting (Ubbens and Stavness, 2017). To segment multiple objects, the instance segmentation model (Romera-Paredes and Torr, 2016) was proposed based on an end-to-end recurrent neural network (RNN). The model designed a spatial attention module to extract small patches, and then uses a convolutional long short-term memory (LSTM) network to build the relation of these patches. By doing so, the model can finish plant leaf segmentation and leaf counting. To solve the target occlusion problem, Ren et al. (Ren and Zemel, 2017) used an RNN-based architecture to generate continuous regions of interest and designed a human-like counting process based on the attention mechanism, thus making it a more accurate segmentation for each object in turn. Lin et al. (Lin et al., 2019) proposed a self-supervised CNN-based framework for leaf segmentation. The model first used self-contained information to classify each pixel, and then the segmentation algorithm for the color leaf images was used to identify the leaf region. Finally, a self-supervised color-based correction model was proposed to segment the complex images taken under complex lighting conditions. As shown in **Table 1**, we summarize the work related to plant leaf segmentation.

**Table 1:** The related works in plant leaf image.

Categories	Author	Method
Conventional method	Kumar and Domnic, 2019	A statistical method with graph-based models
	Wang et al., 2018	The Sobel-based model with green channel information
	Xia et al., 2013	The modified active shape models for plant leaf detection
	Larese et al., 2014	Automatic classification modle for legumes image
	Kuznichov et al., 2019	Augment dataset and the geometrical structure
	Amean et al., 2021	Self-supervised blade segmentation framework
	Lin et al., 2023	Self-supervised semantic segmentation model for complex lighting conditions
Deep learning-based method	Aich and Stavness, 2017	The SegNet-based model for leaves and leaf counting
	Ubbens and Stavness, 2017	A deep learning platform for complex plant phenotyping
	Romera-Paredes and Torr, 2016	Recurrent instance segmentation
	Ren and Zemel, 2017	End-to-end instance segmentation with recurrent attention
	Lin et al., 2019	A self-supervised CNN-based framework for leaf segmentation

It is well-known that U-Net (Ronneberger et al., 2015) is one of the most efficient models and widely used for specific object extraction in image segmentation. U-Net and its variants (Shen et al., 2017) have achieved competitive performance in many computer vision tasks, such as ResU-Net (Zhang et al., 2018), U-Net++ (Zhou et al., 2019), DenseNet (Huang et al., 2017), 3D U-Net (Li et al., 2020), V-Net (Milletari et al., 2016). Bhagat et al. (2022) proposed a modified U-Net architecture for plant leaf segmentation, where an EffcientNet-B4 module was used as an encoder to extract the image feature. Meanwhile, a redesigned skip connection and the residual modules of the decoder were used to reduce computational cost. However, these methods usually ignored the global context information. To be exact, these models could not extract the long-range correlation between pixels, especially for the pixels surrounding the boundary of the objects. The effective method for obtaining the precise location and boundary of the segmentation object was to extract the global context information of the feature map and the long-range correlation between pixels. Transformer has been proved to be an efficient self-attention mechanism to establish long-term dependencies in the field of natural language processing (NLP). More recently, it was introduced into the visual classification tasks. Ramachandran et al. (Ramachandran et al., 2019) explored a novel ResNet-based model by replacing all spatial convolutional layers with the self-attention layers. However, the local self-attention might still lose part of the global structural information. In order to obtain global information of visual images, Vision Transformer (ViT) (Dosovitskiy et al., 2020) inspired by Transformer was proposed to solve the natural image recognition task. ViT first divided the image into several non-overlapping patches, and then used Transformer with the self-attention mechanism to calculate the global information between each token to obtain the global context information. To further reduce the sequence length and computational complexity, Swin Transformer (Liu et al., 2021) used a shifted window to calculate the local self-attention. By establishing a shifted window, two adjacent windows could interact with each other, and cross connections were established between the widows of the upper and lower layers, which improved the effect of global context.

To address these problems, we built a pepper leaf dataset focused on the disease detection segmentation, and propose a bidirectional attention fusion network, named BAF-Net, to obtain the for pepper leaf segmentation. BAF-Net is comprised of three parts: multi-scale fusion feature (MSFF) branch, full-scale feature fusion (FSFF) branch, and bidirectional attention feature fusion (BAF) modules. The backbone of the MSFF branch is a U-shaped network architecture. By incorporating the Swin-Transformer block and the CNN-based module, a cascaded hybrid module (Swin-Trans-Conv) is constructed, to obtain multi-scale fusion features. In the FSFF branch, we first fuse the features of the five-layer encoder from the MSFF branch. Then, the generated features pass through several convolution blocks to obtain the full-resolution feature. The BAF module adaptively fuses the output features of the MSFF and FSFF branches, generating two corresponding features for each branch. In short, the main contributions of our work are as follows:

(1) By incorporating the Swin Transformer and CNN-based modules, we build a cascaded Swin-Trans-Conv block to replace each convolutional layer of U-Net. The Swin Transformer-based module can extract the long-range dependencies while the CNN-based module is used to obtain the local image information.
(2) An FSFF branch is designed to extract detailed information and the boundaries. By incorporating the multi-scale features which are from the outputs of the encoder in the MSFF branch, the boundary information is retained. Meanwhile, the multi-layer full-scale convolution block can extract detailed information.
(3) We propose a BAF module to adaptive share the multi-scale and full-scale features, which can adaptively compute the features of two corresponding branches according to the output features of the MSFF and FSFF branches.
(4) By verifying on four dataset of pepper leaf images, the results show that our model is superior to the state-of-the-art models in terms of the evaluation indices such as IoU and F1score.

The rest of this paper is arranged as follows. Section 2 first reviews the materials including the dataset and its labeling process. Then, the proposed model including the overall architecture, the formulation of the MSFF and FSFF branches, the BAF module, and the loss function are discussed. Finally we introduce the evaluation indices. Section 3 demonstrates the experimental results and discussion. The conclusions are summarized in Section 4.

## 2 Materials and methods

### 2.1 Dataset

In our experiments, the images of pepper leaves were taken from the farm of Nanchang Academy of Agricultural Sciences in Jiangxi Province, China. We took photos for multi-view in the real natural environment from the morning to the afternoon on August 12 and 13, 2021.Pepper leaves were seriously affected by a variety of diseases during growth. Two common diseases of pepper leaf destroyed the normal growth of pepper, such as the brown spot disease and the early blight disease. Meanwhile, we also collect healthy pepper leaves to expand our dataset. As shown in **Table 2**, there are 3921 pepper leaf images in our dataset including the healthy pepper leaves (HPL) and two different categories of infection (2606 images): spot disease (SD) and early blight disease (EBD), and several examples are shown in **Figure 1**. As shown in **Table 2**, the SD, EBD, and HPL datasets contain 1385, 1221, and 1315 images. The total pepper leaf (TPL) dataset is comprised of the SD, EBD, and HPL datasets. In our experiment, the images of each image dataset are split into the training set, the validation set and the test set, and the image numbers of the training set. Meanwhile, in order to evaluate the robustness of the BAF-Net, the images were taken with different complex background as shown in **Figure 1**.

**Table 2:** Four datasets for the validating the proposed model on the pepper leaf.

Dataset	Test	Training	Validation	Total
**Spot Disease (SD)**	186	1015	184	1385
**Early Blight Disease (EBD)**	164	895	162	1221
**Healthy Pepper Leaf (HPL)**	176	965	174	1315
**Total Pepper Leaf (TPL)**	526	2875	520	3921

### 2.2 Dataset labeling

In the following section, we present a data labeling process, and the labeled images are used for validating the proposed model. To accurately annotate the given images, we use the open-source tool named as LabelMe^1^, which was developed by the computer science and artificial intelligence laboratory of MIT university. It allows users to annotate images manually to build image dataset for image segmentation. The pixel-by-pixel way carefully delineated the boundary of each leaf. All these images in the experiment are marked using this tool. Thereafter, each annotated image generates a binary segmentation mask, where the intensity values of the foreground and background are 1 and 0, respectively. During annotating the dataset, we retain the same size as the input image. In view of the computational cost in deep learning, we set the size of the input image to 512×512.

### 2.3 Method

#### 2.3.1 Overall architecture

**Figure 2:**
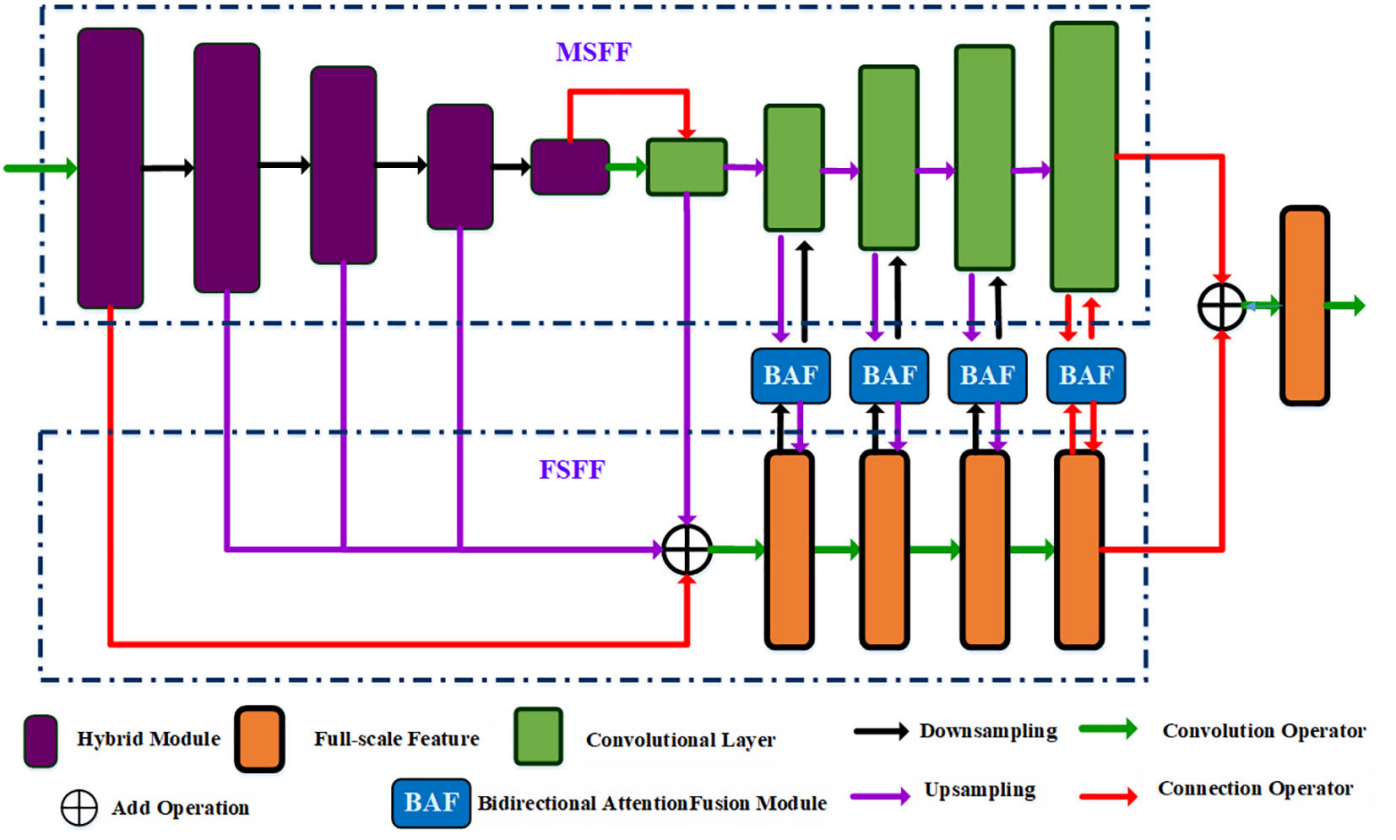
The overall framework of the proposed BAF-Net, which includes three main modules such as the multiscale feature fusion branch, GAM and decoders, where the decoder includes the global context module (GAM) and FAM with LAM.

In the field of image segmentation, U-Net has become one of the most successful network frameworks. It consists of a contracting path and an expanding path, where the contracting path is used to capture the image feature while the expanding path can achieve object localization. In each encoder-decoder layer, a skip connection layer transforms the low-level and high-level information. The model uses a convolution layer with fixed kernel size to extract image features, However, it is difficult to capture long-range semantic information. Although Transformers (Dong et al., 2019) can effectively encode the long-range dependencies, it is difficult to obtain local details and accurate boundaries of pepper leaves. To solve this problem, we propose a bidirectional attention fusion network by combining CNN and Transformer for pepper leaf segmentation, also named as BAF-Net, where CNN is used to extract the local image information while the Transformer-based module can capture the long-range dependencies. As shown in **Figure 2**, the multi-scale branch is used to extract the global features while the full-scale feature can retain the detailed boundary information. The bidirectional fusion module is designed to concatenate the multi-scale features and the full scale features.

Specifically, BAF-Net includes three parts: a multi-scale feature fusion (MSFF) branch, a full-scale fusion feature (FSFF) branch, and bidirectional attention fusion (BAF) modules. In the MSFF branch, the network structure is similar to U-Net, composed of an encoding path and a decoding path. Different from the U-Net model, the encoder is replaced by a hybrid module by incorporating the convolutional layer and the Swin Transformer (Liu et al., 2021) module, and the decoder is composed of convolutional modules. In the FSFF branch, we first upsample four features: the output features of the encoder from the 2^nd^ layer to the 4^th^ layer, and the 5^th^ layer of the decoder. Four output features are the same size as the first layer’s output feature in the MSFF branch. Then, we fuse five generated features, and the generated feature is passed through four continuous convolutional modules. Each convolutional module is activated by the convolution layer, batch normalization, and the ReLU activation function. In the BAF module, the input features are from the output feature of the decoder in the MSFF branch and the output feature of the corresponding convolutional module in the FSFF branch. By incorporating the MSFF and FSFF branches, the improved model not only achieves the full resolution feature but also extracts the comprehensive and multi-scale features.

#### 2.3.2 Multi-scale feature fusion branch

**Figure 3:**
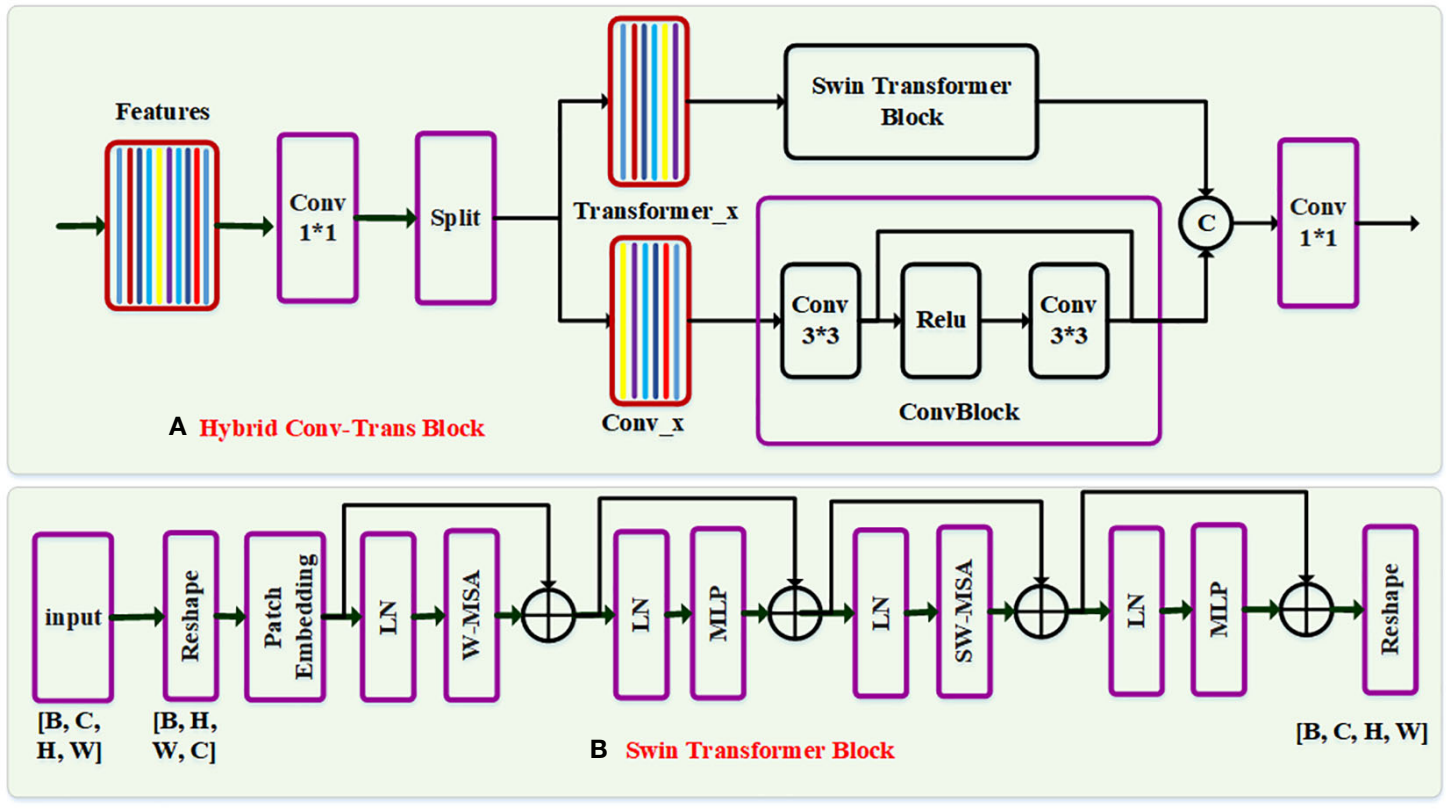
The network structure of the Swin-Trans-Conv block. In each block, the input feature is first passed through a 1×1 convolution, and subsequently is split evenly into two feature map groups, each of which is then fed into a Swin transformer block and a residual 3×3 convolutional (RConv) block, respectively. Afterwards, the output features of the Swin-Trans-Conv block and the RConv block are concatenated and then passed through a 1×1 convolution to generate a novel feature *via* a residual path.

The transformer-based model (Dosovitskiy et al., 2020; Cao et al., 2021) has a more robust representation than the CNN-based model while building the long-range dependencies. In order to extract the global features, we explore a hybrid Swin-Trans-Conv block by combining the Swin-Transformer encoder and the convolutional layer, which is used to replace the convolutional layer of the encoder in the MSFF branch. As shown in **Figure 3A**, the backbone network including an encoder network and a decoder network is similar to U-Net. In the encoder network, we use a hybrid module by combining the convolutional layer and the Swin Transformer block, also called as Swin-Trans-Conv block, to replace each convolutional layer of U-Net, where an average pooling operator perform the downsampling process and the size of the feature maps are changed into half of the original. The decoder network is comprised of four convolutional layers and four upsampling operators. The upsampling operation is achieved by performing a deconvolutional operator with the stride of 2. The convolutional layer consists of a convolutional operator, batch normalization, and a ReLU activation layer. The number of channels in five layers corresponding to the 1^st^ layer to the 5^th^ layer is 32, 64, 128, 256 and 512, respectively.

Assuming that the input feature is X∈*R* ^B×H×W×C^, where B, C, H and W represent the batch size, the channel number, and the image height and width of the input feature, respectively. In the Swin-Trans-Conv block as shown in **Figure 3A**, we first transforms the input feature X into X’∈*R* ^B×H×W×C^. Then, we perform a 1×1 convolution operator on the generated feature, and split the generated feature Y into two groups F*_Trans_* and F*_conv_*, which can be expressed as:


(1) Y=Conv1×1(Reshape(X))                                           X,Y∈RB×C×H;×W

(2) $${\text{F}}_{trans},\;{\text{F}}_{conv}=\text{Split}(Y)\;{\text{F}}_{Trans}{\text{,F}}_{conv}\in {\text{R}}^{B\times C/2\times H\times \text{W}}$$

where Reshape(·) is a reshape operator on two feature matrix F*_trans_* and F*_conv_*, ${\text{Conv}}_{1\times 1}\left(\cdot \right)$ denotes a 1×1 convolutional operator, and Split(·) represents a split operation on the multidimensional matrix. Finally, the feature F*_Trans_* is passed through a module based on Swin Transformer (Swin-Trans) encoder, and the generated feature map ${\text{F}}_{trans}^{'}$ is written as:


(3) $${\text{F}}_{trans}^{'}=\text{SwinTrans}({\text{F}}_{trans})$$

Similarly, the feature map F*_conv_* passes through a residual convolution module, and the generated feature ${\text{F}}_{conv}^{'}$ is defined as:


(4) $${\text{F}}_{conv}^{'}=\text{RConv}({\text{F}}_{conv})$$

where RConv(·) is the residual convolution module, which is comprised of a 3×3 convolution filter, a ReLU activation layer, and a 3×3 convolution filter by a residual path, which is rewritten as:


(5) $${\text{F}}^{3}={\text{Conv}}_{3\times 3}({\text{F}}_{conv})$$

(6) Fconv'=Conv3×3(Relu(F3))+F3

where F^3^ is the feature map performed a 3×3 convolution operation on the feature map F*_conv_*, Conv_3×3_ is a 3×3 convolutional layer, and Relu(·) is a ReLU activation layer. Finally, we concatenate two features ${\text{F}}_{trans}^{'}$ and ${\text{F}}_{conv}^{'}$ , and then perform a 1×1 convolution filter by a residual path, which is represented as:


(7) $$X_{out}={\text{Conv}}_{1\times 1}\left({\text{F}}_{trans}^{'}⊙{\text{F}}_{conv}^{'}\right)+X$$

where ⊙ denotes the concatenation operation.

Meanwhile, to construct the Swin-Trans module as shown in **Figure 3B**, the input feature F*_Trans_* is split into small patches, and each patch size is set to *P* × *P* × *P*, where P is a positive integer and the number of the patches is *S*=[*H*/*P*]×[*W*/*P*]×[*C*/*P*]. For the feature ${\text{F}}_{Trans}^{i}$ of the *i*-th layer with the 3D patches, we first compute the multi-head self-attention in a small window (W-MSA), which can be formulated as:


(8) $${\hat{F}}_{out}^{i}=\text{W-MSA}\left(\text{LN}({\text{F}}_{Trans}^{i})\right)+{\text{F}}_{Trans}^{i}$$

(9) $${\hat{F}}_{mlp}^{i}=\text{MLP}\left(\text{LN}({\hat{F}}_{Out}^{i})\right)+{\hat{F}}_{Out}^{i}$$

where W-MSA(·) denotes the window multi-head self-attention, LN(·) is the layer normalization operator, and MLP(·) denotes a multilayer perceptron module with two fully-connected layers and the GELU activation function. Then, the generated feature ${\hat{F}}_{mlp}^{i}$ is passed through the multi-head self-attention in the shifted window (SW-MSA), which is represented as:


(10) $${\hat{F}}_{sw}^{i}=\text{SW-MSA}\left(\text{LN}({\hat{F}}_{mlp}^{i})\right)+{\hat{F}}_{mlp}^{i}$$

(11) $${\text{F}}_{out}^{i}=\text{MLP}\left(\text{LN}({\hat{F}}_{sw}^{i})\right)+{\hat{F}}_{sw}^{i}$$

where SW-MSA(·) denotes the shifted window multi-head self-attention, and ${\text{F}}_{out}^{i}$ is the output feature of the *i*-th layer. Finally, the output feature ${\text{F}}_{out}^{i}$ is reshaped into the same size of the input feature in the Swin-Trans module.

It is worth noting that the Swin-Trans-Conv block has several advantages. First, it integrates the local modeling capability of the convolution module and the global modeling capability of the Swin-Trans module. Secondly, the split and concatenation operations are used for two branches to extract different features, reducing the computational complexity and the number of parameters.

#### 2.3.3 Full-scale feature fusion branch

The edge and detailed image information may be lost in the U-shape network framework due to the continuous downsampling operators. To solve this problem, we design an MSFF branch to retain the detailed information, and the network structure is shown in **Figure 2B**. We fuse the output features of the first 1^st^ to 4^th^ layer in the encoder of the MSFF branch and the output feature of the decoder of the 5^th^ layer in the decoder since the multi-scale features can enhance the edge information (Liu et al., 2023). For four output features from the MSFF branch, we first carry out a 1×1 convolution filter to reduce the channel number. Then, we perform the upsampling operator on the four features, and the four generated features have the same size with the first channel feature. Then, we integrate four generated features into the input feature by a residual path, which can be expressed as:


(12) $${\text{X}}_{i}^{up}={\text{Conv}}_{1\times 1}\left(\underset{i-1}{\underset{︸}{\text{up}(\cdots \text{up}({\text{X}}_{i}))}}\right)\;i=2,\cdots ,5$$

(13) $${\text{X}}_{fuse}=\sum_{i=2}^{5}{\text{X}}_{i}^{up}+\text{X}$$

where Conv_3×3_ denotes a 3×3 convolutional filter.

Finally, the novel feature passes through four continuous convolutional modules. Each convolutional module includes a 3×3 convolution layer, batch normalization, and a ReLU activation layer. To reduce the computational cost and the parameters, we keep each channel number of four features equal to that of the first layer in the MSFF branch. In this paper, the channel number is set at 32. The operations for each convolution block are presented as follows:


(14) {Xf4=Relu(BN(Conv3×3(ffuse) ) )                                                         i=5  Xfi−1=Relu(BN(Conv3×3(Xfi)))                                    i=2,  ⋯,  4

#### 2.3.4 Bidirectional attention fusion module

**Figure 4:**
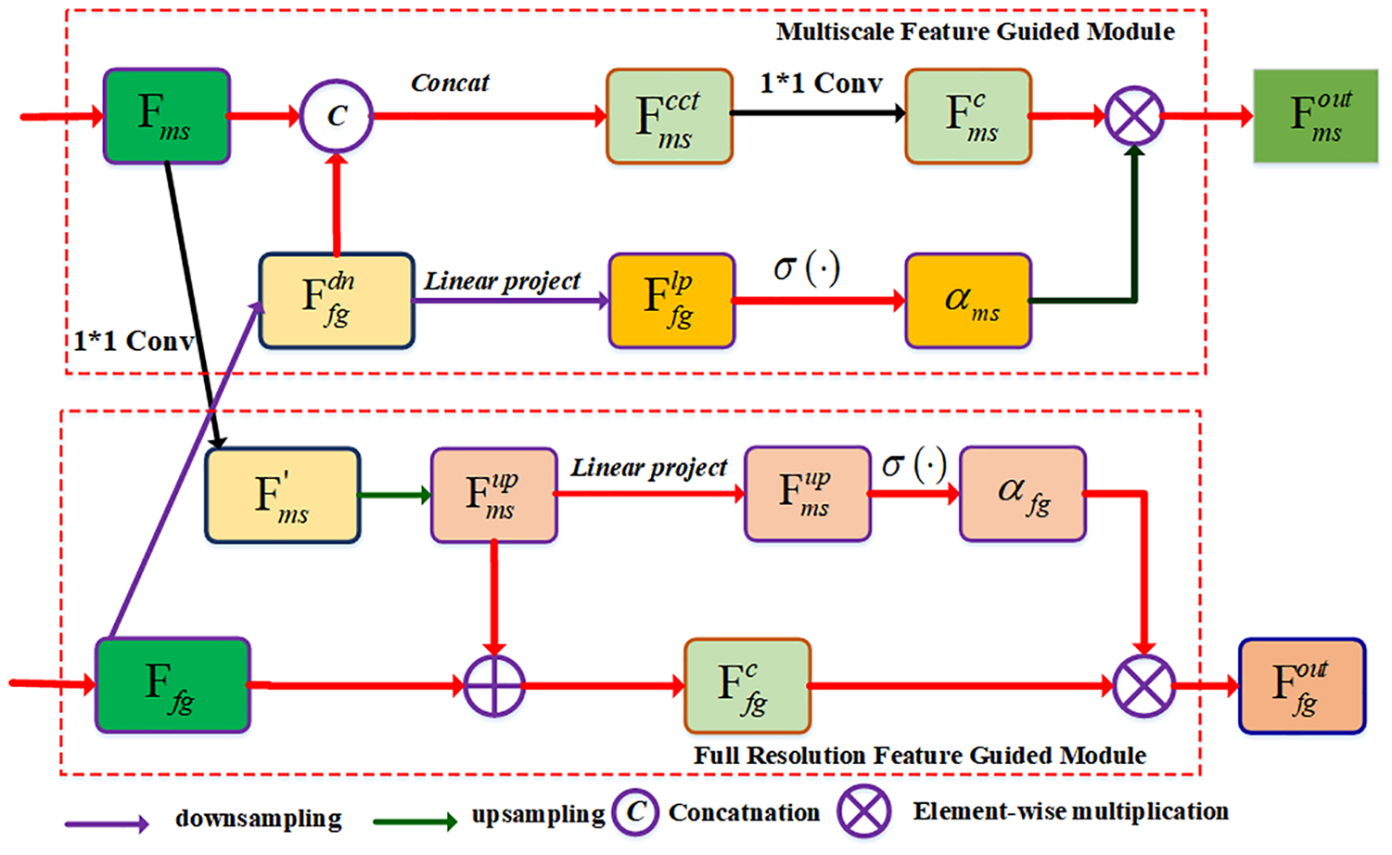
The network structure of the BAF module. Two input features F*_ms_* and F*_fg_* are from the output features of the MSFF and FSFF branches, respectively. The BAF module contains a multi-scale feature guided (MSFG) module and a full resolution feature guided (FRFG) module. The MSFG module is used to generate the multi-scale feature while the FRFG module is used to generate the full-scale feature.


In order to achieve the multi-scale and full-scale features, we designed a BAF module to generate the corresponding output features for the MSFF and FSFF branches. As shown in **Figure 4**, the BAF module includes multi-scale feature guidance (MSFG) module and full-scale feature guidance (FSGM) module. For the MSGM module, we first conduct the downsampling operation on the input feature of the FSGM module, and the novel feature maps have the same spatial dimensions with the same with that of the MSGF map, which can be expressed as:

(15) $${\text{F}}_{fg}^{dn}=\text{DN}\left({\text{F}}_{fg}\right)$$

where DN(·) denotes the downsampling operation. Then, we concatenate the output feature of the MSFF branch F*_ms_* and the feature ${\text{F}}_{fg}^{dn}$ , and perform a 1×1 convolution module on the novel feature map to compress the number of channels, we can obtain the feature map:


(16) $${\text{F}}_{ms}^{c}={\text{Conv}}_{1\times 1}\left({\text{F}}_{ms}\text{e}{\text{F}}_{fg}^{dn}\right)$$

At the same time, we project the feature ${\text{F}}_{ms}^{c}$ to compress the feature map into a channel along the channel direction, and use the Sigmoid activation function to obtain the global attention map, which is defined as:


(17) $$\alpha_{ms}=\sigma_{sig}\left(\mathrm{Pr}\text{oj}\left({\text{F}}_{ms}^{c}\right)\right)$$

where Proj(·) denotes the linear projection function, $\sigma_{sig}\left(\cdot \right)$ denotes the Sigmoid function, and α*_ms_* ∈[0,1] is the spatial attention map of the feature ${\text{F}}_{ms}^{c}$ . It is obvious that the spatial attention map α*_ms_* calculates the spatial weight of each pixel, and the calibrated feature map is expressed as:


(18) $${\text{F}}_{ms}^{out}=\alpha_{ms}⊗{\text{F}}_{ms}^{c}$$

Finally, the feature ${\text{F}}_{ms}^{out}$ is transformed to the next convolution layer.

In the FSFG module, we first perform a 1×1 convolutional filter on the multi-scale feature map F*_ms_* to compress the number of channels. The expression is as follows:


(19) $${\text{F}}_{ms}^{'}={\text{Conv}}_{1\times 1}\left({\text{F}}_{ms}\right)$$

Then, we upsample the multi-scale feature map to make the generated features have the same spatial dimension as that of the full-scale feature. The expression is as follows:


(20) $${\text{F}}_{ms}^{up}=\text{up}\left({\text{F}}_{ms}^{'}\right)$$

where up(·) denotes the upsampling operator. Afterwards, two features ${\text{F}}_{ms}^{up}$ and F*_fg_* are fed into the convolutional layer to generate a new feature ${\text{F}}_{fg}^{c}$ , which is written as:


(21) $${\text{F}}_{fg}^{c}={\text{Conv}}_{3\times 3}\left({\text{F}}_{ms}^{up}⊕{\text{F}}_{fg}\right)$$

where ⊕ represents the pixel-wise addition operation. Meanwhile, we use linear projection to compress the feature into a channel along the channel direction, and then use the sigmoid activation function to obtain the global attention map:


(22) $$\alpha_{fg}=\sigma_{sig}\left(\mathrm{Pr}\text{oj}\left({\text{F}}_{fg}^{c}\right)\right)$$

where *α_fg_* ∈[0, 1] is the spatial attention map of ${\text{F}}_{fg}^{c}$ , which is used to calculate the spatial position weight of each pixel. The calibrated feature map can be represented as:


(23) $${\text{F}}_{fg}^{out}=\alpha_{fg}⊗{\text{F}}_{fg}^{c}$$

Finally, it is input to the convolution layer of the next FSFG module.

#### 2.3.5 Training loss

The network should be trained to obtain the best training parameters. It is known that the loss function is essential to the predicted performance of the segmentation model. The loss function is used to measure the deviation between the model prediction and the ground truth. The binary cross entropy (BCE) is a loss function widely used in binary image segmentation tasks. Assuming that the input predicted result is *p*, and the corresponding ground truth label is *g*, the BCE loss function is defined as:


(24) $$L_{bce}(p,\;g)=-\sum_{i=1}^{N}\left[g_{x}\log(p_{x})+(1-g_{x})\log(1-p_{x})\right]$$

The intersection over union (IoU) loss is defined as:


(25) $$L_{IoU}(p,\;g)=-\log\left(\frac{\sum_{i=1}^{N}\left|g_{x}\cdot p_{x}\right|}{\sum_{i=1}^{N}\left(g_{x}+p_{x}-\left|g_{x}\cdot p_{x}\right|\right)}\right)$$

Therefore, our final loss includes *L_bce_* and *L_IoU_*, which can be expressed as:


(26) $$L_{total}(p,\;g)=\alpha L_{bec}(p,g)+(1-\alpha )L_{IoU}(p,g)$$

The weight α is a coefficient to balance the importance of two loss functions, and we set *α*=0.5.

### 2.4 Performance evaluation

In order to verify the segmentation performance, we use six evaluation indices to evaluate the accuracy of the model on the pepper leaf datasets. Six evaluation indices include: pixel accuracy (PA), pixel recall (PR), pixel precision (PP), pixel specificity (PS), intersection over union (IoU) and F1 score. We assume that TP (True Positive) represents the number of pixels that are both 1 in the predicted value and the label value, TN (True Negative) represents the number of pixels that are both 0 in the predicted value and the label value, FP (False Positive) represents the number of pixels that are 1 in the predicted value and 0 in the label value, and FN (False Negative) represents the number of pixels that are 0 in the predicted value and 1 in the label value. The expression of the pixel accuracy is written as follows:


(27) $$PA=\frac{TP+TN}{TP+TN+FP+FN}$$

PR is defined as follows:


(28) $$PR=\frac{TP}{TP+FN}$$

PP is defined as follows:


(29) $$PP=\frac{TP}{TP+FP}$$

F1 score is defined as:


(30) $$F1=\frac{2\times PR\cdot PP}{PR+PP}$$

PS is defined as follows:


(31) $$PS=\frac{TN}{TN+FP}$$

From Equations (27)-(31) and the IoU as defined in Equation(25), it can be seen that six evaluation indices range from 0 to 1. The higher the index values are, the best segmentation performance is obtained. Generally speaking, the mean IoU (mIoU) is used to evaluate the segmentation performance on a given dataset.

## 3 Experiments

In this section, we present the experimental results including the experimental settings, the comparison with the state-of-the-arts models, the ablation study and the discussion.

### 3.1 Experimental settings

All models in the experiment are carried out on Intel (R) Core (R) i7-8700K CPU 3.70GHz CPU and Nvidia GeForce TITAN XP 12 GB GPU with 48G RAM. The programs are conducted on the Ubuntu 16.04 with the Conda environment. In the BAF-Net, the parameter settings are as follows: the batch size is set to 4, the number of iterations (epoch) is set to 60, and each epoch contains 350 batches. During the training process, the network is optimized using stochastic gradient descent (SGD), the initial learning rate is set to 0.01.

### 3.2 Comparison with the state-of-the-arts models

We compared BAF-Net with the state-of-the-art methods on four pepper leaf datasets, such as the SD, EBD, HPL, and TPL datasets. For fairness, these models are running on the same training dataset, the validation dataset, and the test dataset. The comparative models on the pepper leaf dataset involve U-Net (Ronneberger et al., 2015), AttU-Net (Oktay et al., 2018), Swin-UNet (Cao et al., 2021), SCUNet (Zhang et al., 2022) and the proposed BAF-Net. We set the training epochs to 60 for each trained model.

**Figure 5:**
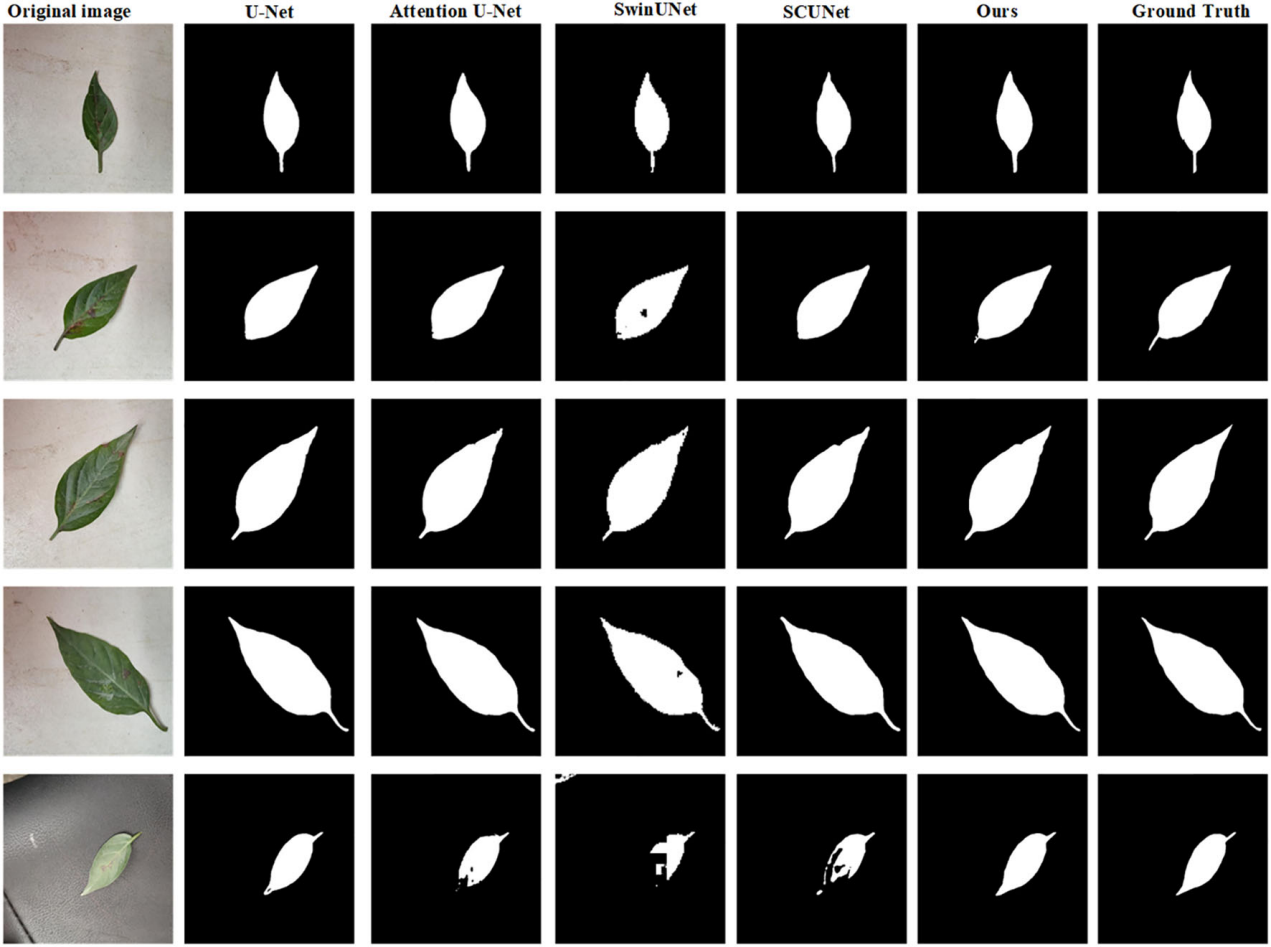
Examples of the predicted results using five different models on the SD dataset. From the 1^st^ column to 7^th^ column: the original images, the predicted results using the U-Net, attention U-Net, Swin-UNet, SCUNet, the proposed model and ground truth (GT), respectively.

**Table 3** shows the test results on the SD dataset using five different state-of-the-art models. Compared with U-Net, the proposed model has a precision increase of 7.48%, IoU increase of 3.88%, and F1 score increase of 5.0%. It also shows that PA score has the relative improvement of 0.5% on the SD dataset. For the attention U-Net model, the segmentation results on five indices are close to that of the U-Net. In addition, Swin-UNet and SCUNet have the similar segmentation performance. However, the segmentation performance of U-Net exceeds two models in terms of six evaluation indices. The reason is that Swin-UNet and SCUNet containing the transformer-based modules attain better segmentation results only if more efficient pre-trained model is provided. From **Table 3**, where the highest score for each indicator is shown in bold, our model can obtain the best segmentation performance in terms of five evaluation indices including PA, PP, PS, IoU, and F1 scores compared with other models. By evaluating the segmentation performance of five models, we also give several examples of the segmentation results using these compared methods as shown in **Figure 5**.

**Table 3:** The segmentation results on the SD dataset using five different models.

Model	PA(%)	PR(%)	PP(%)	PS(%)	mIoU(%)	F1(%)
**UNet**	98.70	**99.09**	91.10	98.65	91.80	94.93
**Attention U-Net**	98.24	98.11	88.72	98.26	90.09	93.18
**Swin-UNet**	97.68	97.90	85.36	97.65	86.76	91.20
**SCUNet**	97.42	98.14	83.68	97.32	87.34	90.33
**Ours**	**99.20**	98.74	**98.58**	**99.80**	**95.68**	**96.75**

**Figure 6:**
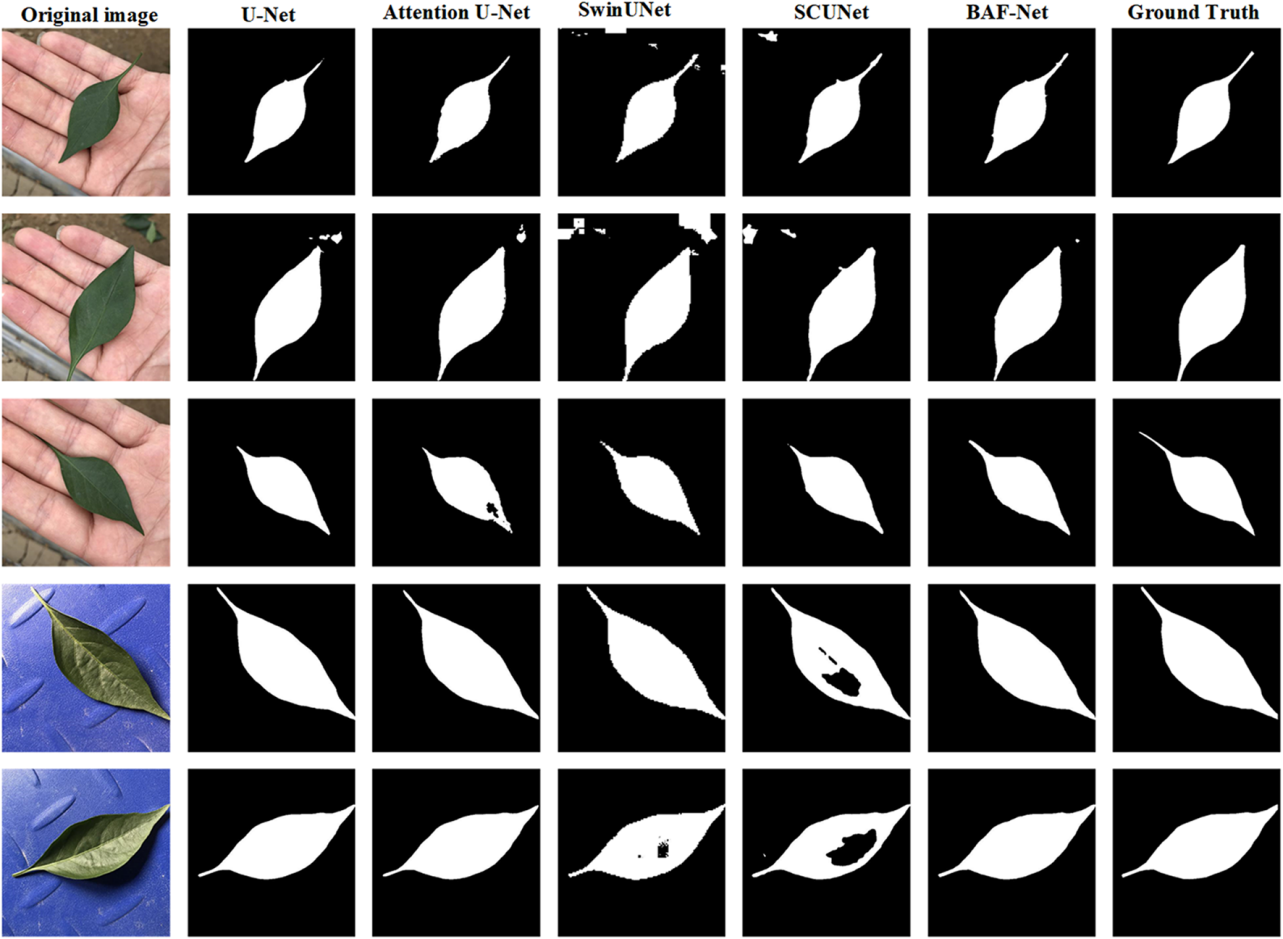
Examples of the predicted results using five different model on the EBD dataset. From the 1^st^ column to 7^th^ column: the original image, the predicted results using the U-Net, attention U-Net, Swin-UNet, SCUNet, and our model, respectively.

**Table 4** presents the segmentation results of five segmentation models on EBD pepper leaf dataset, in which the highest score of each index is shown in bold. From the experimental results, the proposed model has the highest scores among the six indices including PA, PR, PP, PS, IoU and F1 scores. Specifically, compared with U-Net, BAF-Net increased PA by 0.62%, PR by 0.06%, PP by 3.44%, PS by 1.7%, IoU by 5%, and F1 score by 7.04%. Attention U-Net is only lower than BAF-Net in terms of the indices IoU and F1-score, with a decrease of 4.92% and 6.59%, respectively. Compared with Swin-Unet and SCUNet, the proposed model has significant improvement in terms of six indices. The proposed BAF-Net have significant improvement in terms of PP, reaching the increase by 7.25% and 14.29%, respectively. By evaluating the segmentation performance of five deep learning-based models, we find these models can obtain better segmentation results than the traditional methods. Meanwhile, we also give the examples of the segmentation results using these compared methods as shown in **Figure 6**.

**Table 4:** The segmentation results on the EBD dataset using different models.

Model	PA(%)	PR(%)	PP(%)	PS(%)	mIoU(%)	F1 (%)
**UNet**	96.98	98.11	81.54	95.82	81.76	84.06
**Attention U-Net**	95.57	96.63	75.08	95.41	81.84	84.51
**Swin-Unet**	96.02	95.55	77.73	96.08	80.63	85.72
**SCUNet**	94.49	95.70	70.69	94.32	77.33	81.31
**BAF-Net(ours)**	**97.60**	**98.17**	**84.98**	**97.52**	**86.76**	**91.10**

**Figure 7:**
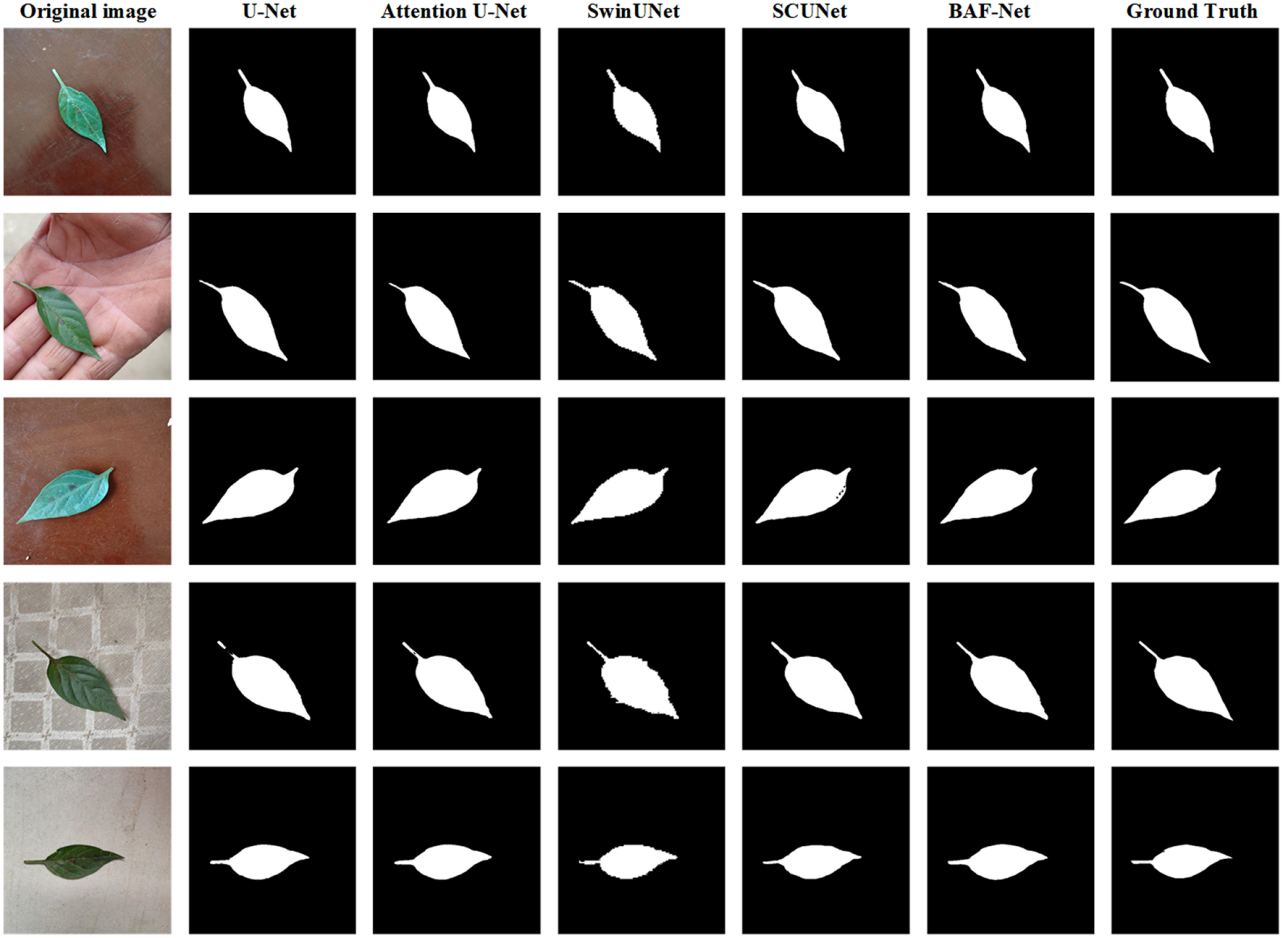
Examples of the predicted results using five different model on the HPL dataset. From the 1^st^ column to 7^th^ column: the original image, the predicted results using the U-Net, attention U-Net, Swin-UNet, SCUNet, and our model, respectively.

**Table 5** shows the validation results of five different models on the HPL data set, with the highest score for each indicator shown in bold. From the experimental results, the proposed model can obtain the best segmentation accuracy in terms of PA, PP, PS, mIoU and F1 score. Compared with U-Net, the proposed model has increased PA by 0.21%, PP by 1.51%, PS by 1.52%, IoU by 0.01%, and F1 score by 0.27%. The attention U-Net has the similar segmentation results with U-Net. Our model has significant improvement than Swin-UNet and SCUNet in terms of the PP, mIoU and F1 score. Compared with the Swin-UNet, the PP, mIoU and F1 scores have increased by 4.95%, 3.60% and 2.43%, respectively. Compared with the SCUNet, the PP, mIoU and F1 score has increased by 2.51%, 2.22% and 1.58%, respectively. Meanwhile, we also give the example of the segmentation results for qualitative comparison, and the representative examples are shown in **Figure 7**.

**Table 5:** The segmentation results on the HPL dataset using different models.

Model	PA(%)	PR(%)	PP(%)	PS(%)	mIoU(%)	F1 (%)
**U-Net**	99.09	**98.51**	95.84	99.02	96.11	97.07
**Attention U-Net**	99.21	97.69	96.34	99.44	95.53	97.01
**Swin-UNet**	98.63	97.57	92.40	98.79	92.52	94.91
**SCUNet**	98.88	96.69	94.84	99.21	93.90	95.76
**BAF-Net(ours)**	**99.30**	97.32	**97.35**	**99.60**	**96.12**	**97.34**

The experimental results on the TPL dataset are shown in **Table 6**, with the highest score in each indicator represented in bold. It can be seen that our model obtains the best segmentation results in terms of the six indices among five models. Compared with U-Net, the proposed model has IoU increased by 0.01%, PA increased by 0.13%, PR increased by 0.03%, and PP increased by 0.87%. The PS score is 0.01% higher than that of U-Net, and F1 score is 0.48% higher than that of U-Net. Compared with attention U-Net, the proposed model has significant improvement in terms of six indices. However, Swin-UNet and SCUNet do not improve the segmentation results compared with the U-Net. In summary, BAF-Net has obvious advantages in segmenting the pepper leaf from the natural images.

**Table 6:** The segmentation results on the pepper leaf dataset using different models.

Model	PA(%)	PR(%)	PP(%)	PS(%)	mIoU(%)	F1 (%)
**U-Net**	98.40	98.59	89.70	98.37	91.43	93.94
**Attention U-Net**	97.73	97.51	86.28	97.76	89.31	91.55
**Swin-UNet**	97.48	97.06	85.07	97.54	86.87	90.67
**SCUNet**	97.00	96.88	82.40	97.01	86.32	89.06
**BAF-Net(ours)**	**98.53**	**98.62**	**90.57**	**98.52**	**91.44**	**94.42**

### 3.3 Ablation study

In this section, we perform an ablation study to validate the effectiveness of each module. Especially, we consider the basic U-Net architecture as the baseline, namely the simple U-Net (SU-Net), which is similar to U-Net with half of the channel number of U-Net. In the ablation experiments, we take SU-Net, MFF, MRF, and BAF as four basic modules. Our experimental strategy is to add a module each time, and it is proven to be effective. We approve that it is effective in subsequent studies. Strictly speaking, we selected four unique models, such as SU-Net, SU-Net-MFF, SU-Net- MFF-MRF, and BAF-Net, to verify that different modules are still valid when each model is added to SU-Net each time.

As shown in **Table 7**, we first experiment SU-Net-MSFF by replacing the convolution layer of the encoder in the SU-Net model with the Swin-Trans-Conv block, which is formulated by adding the MSFF module into SU-Net. Experiments show that PA, PR, PP, PS, mIoU and F1 score of the SU-Net-MSFF model are 98.94%, 96.87%, 96.90%, 99.36%, 95.63% and 96.88, respectively. Then, by adding the FSFF module to SU-Net-MSFF, the results show that the PA, PA, PP, PS, IoU and F1 scores of the SU-Net-MSFF-FSFF are 98.94%, 96.62%, 97.14%, 99.42%, 95.68% and 96.98%, respectively. Compared with the SU-Net-MSFF, PR, PS, IoU and F1 score of the SU-Net-MSFF-FSFF model are increased by 0.24%, 0.06%, 0.05% and 0.10%, respectively. Finally, we experiment BAF-Net by fusing the output features of the decoder in the MSFF and FSFF branches to the BAF modules. The results show that PA, PR, PP, PS, IoU and F1 score of BAF-Net are 98.98%, 6.82%, 97.20%, 99.43%, 95.86% and 97.01%, respectively, which are increased by 0.04%, 0.2%, 0.06%, 0.01%, 0.18% and 0.03%, respectively. From the segmentation results, we can see that the addition of the Swin-Trans-Conv block expands the receptive field and enhances the feature extraction ability of SU-Net, enabling it to obtain different levels of information at the same time. The full-resolution features enable the proposed model to retain image local details. By combining multi-scale information and full scale information, it can extract deeper structural information. Therefore, the combination of the three modules can obtain the best performance.

**Table 7:** Comparison of pepper segmentation results of four models on the dataset.

Model	MSFF	FSFF	BAF	PA%	PR%	PP%	PS%	IoU%	F1%
**SU-Net**				98.86	96.52	96.80	99.35	95.35	96.66
**SU-Net-MSFF**	**√**			98.94	**96.87**	96.90	99.36	95.63	96.88
**SU-Net_MSFF-FSFF**	**√**	**√**		98.94	96.62	97.14	99.42	95.68	96.98
**BAF-Net**	**√**	**√**	**√**	**98.98**	96.82	**97.20**	**99.43**	**95.86**	**97.01**

### 3.4 Discussion

The above analysis shows that the segmentation results of these deep learning-based segmentation models are suitable. Compared with the classical methods based on the variational statistics theory (Costa et al., 2019; Fang et al., 2019a; Fang et al., 2019b; Gao and Lin, 2019; Liu et al., 2020; Fang et al., 2021a; Fang et al., 2021b; Liu et al., 2021; Ward et al., 2021), the deep-learning-based models can obviously obtain better classification results. In our work, to capture the long-range dependencies between different pixels, we propose a bidirectional adaptive attention fusion network called BAF-Net by exploring an adaptive attention mechanism to extract multi-scale and full-scale features simultaneously. Specifically, we first design an MSFF branch based on the encoder-decoder structure, which can not only extract local information of the target, but also learn the spatial attention to increase the receptive field. To further retain the boundary information of the segmented object, we propose a FSFF branch, and design adaptive bidirectional attention modules to achieve the bidirectional connection between the MSFF module and the FSFF module.

The results of the ablation experiment in **Table 7** shows that progressive network such as SU-Net, SU-Net-MSFF, SU-Net-MSFF-FSFF and BAF-Net can improve the predicted performance of the baseline (SU-Net). Compared with the baseline, three models by progressively adding the MSFF, FSFF and BAF modules increase mIoU by 0.28%, 0.33% and 0.51%, respectively, and F1 score increased by 0.02%, 0.02% and 0.36%, respectively. From the segmentation results, it can be seen that BAF-Net has achieved the best performance. Compared with the baseline, the mIoU and F1 score of BAF-Net reaches 95.86% and 97.01%, respectively.

Although the proposed BAF-Net can obtain better performance on the four pepper leaf datasets, there are disadvantages in this work. (1) In the training process, the epoch number in our model is set to 60. Therefore, we need explore a schema to stop the training process for the deep learning-based model automatically. (2) Our model is supervised learning, which requires many training samples. Accordingly, in our future work, we will focus on the semi-supervised or self-supervised segmentation methods to reduce the requirements for training samples.

## 4 Conclusion

In our work, we propose a bidirectional adaptive attention fusion network for automatic segmentation of pepper leaves. The proposed model consists of the MSFF branch with the like-U-Net network structure, the FSFF branch, and the BAF modules with an adaptive attention mechanism. This MSFF branch fuses the Swin-Transformer-based and CNN-based modules to construct the Swin-Trans-Conv block, which replaces the convolution layer of the encoder of U-Net to expand the receptive field. In the MSFF branch, the CNN-based layer can extract the local image features while the Swin-Transformer-based module is used to extract the long-range dependencies of the channel and spatial information to expand receptive field. The FSFF branch performs multiple convolution layers keeping the same size with the original image, which is used to retain the boundary information and detail information of the segmented object. In addition, the BAF modules are used to fuse the output features of the MSFF and FSFF branch, which output the corresponding features for each branch. Compared with the existing model, our model obtain the highest evaluation indices on four pepper leaf datasets. In addition, the ablation experiment shows that the proposed three modules including MSFF, FSFF and BAF are effective. In the future, we will explore a weak-supervised model for pepper leaf segmentation since the small dataset may cause over-segmentation. Meanwhile, we study the construction of loss function and the method for augmentation dataset.

## Data availability statement

The original contributions presented in the study are included in the article/supplementary material. Further inquiries can be directed to the corresponding author.

## Author contributions

JF: conceptualization, methodology, experiment, and writing. HL: supervision and writing- review & editing. HJ: experiment. YF: methodology and investigation. SZ: methodology and investigation. LS: experiment. XH: writing-review & editing. JL: review & editing. MG: review & editing. All authors contributed to the article and approved the submitted version.

## Footnotes

1
https://github.com/wkentaro/labelme
